# The Stone Moroko *Pseudorasbora parva* Altered the Composition and Stability of Sediment Microbial Communities Within the Chinese Mitten Crab (*Eriocheir sinensis*) Polyculture Pond

**DOI:** 10.3390/biology14091297

**Published:** 2025-09-19

**Authors:** Yiran Hou, Yun Bao, Rui Jia, Linjun Zhou, Lili Song, Baojuan Yang, Bing Li, Jian Zhu

**Affiliations:** 1 Key Laboratory of Integrated Rice-Fish Farming Ecology, Freshwater Fisheries Research Center, Chinese Academy of Fishery Sciences, Ministry of Agriculture and Rural Affairs, Wuxi 214081, China; houyr@ffrc.cn (Y.H.); byshou@outlook.com (Y.B.); jiar@ffrc.cn (R.J.); zhoulinjun@ffrc.cn (L.Z.); songlili@ffrc.cn (L.S.); yangbaojuan@ffrc.cn (B.Y.); 2 Wuxi Fisheries College, Nanjing Agricultural University, Wuxi 214081, China

**Keywords:** river crab polyculture, stone moroko, *Pseudorasbora parva*, pond sediment, bacterial, fungal, and protistan communities

## Abstract

Integrated aquaculture, centered around polyculture involving multiple species, is a typical practice for the sustainable development of the aquaculture industry, capable of enhancing resource utilization efficiency, environmental stability, and overall productivity through establishing symbiotic interactions among species. This study employed multi-amplicon high-throughput sequencing to assess the ecological impacts of two polyculture methods involving river crabs on sediment bacteria, fungi, and protists. One method involved polyculturing river crabs with mandarin fish, silver carp, and the stone moroko (SPC), and the other involved polyculturing river crabs with only mandarin fish and silver carp (SMC). The results showed that, compared to the SMC group, the SPC group remarkably increased the Chao1 index of bacterial communities in pond sediment and decreased the Pielou_J index of protists. The relative abundances of all fungal phyla and most dominant bacterial and protistan phyla (top 10 in relative abundance) in the SPC group were considerably different from those in the SMC group. In the co-occurrence networks of bacterial, fungal, and protistan communities, the numbers of edges and nodes were higher in the SPC group than in the SMC group, and the habitat niche breadth of bacterial community was also notably increased in the SPC group. The levels of total carbon (TC), total nitrogen (TN), and phosphates within pond sediment in the SPC group were obviously lower than those in the SMC group, and were significantly correlated with the microbial communities, with TC being identified as the primary contributor driving changes in the microbial communities. All the findings collectively demonstrate that the polyculture of river crabs with mandarin fish, silver carp, and the stone moroko enhances the stability of bacterial, fungal, and protistan communities in sediment and enhances resource utilization efficiency in aquaculture, thereby preventing the environmental risks associated with excessive nutrient accumulation in sediment. Polyculture systems integrating river crabs with mandarin fish, silver carp, and the stone moroko represent a sustainable aquaculture model with significant ecological benefits.

## Simple Summary

The Chinese mitten crab (*Eriocheir sinensis*) holds significant economic value in China, with its aquaculture production in 2023 accounting for about 16.7% of the nation’s total freshwater crustacean production. To maintain a good aquaculture environment and enhance productivity, the primary production pattern for river crabs is through polyculture in ponds, where the silver carp (*Hypophthalmichthys molitrix*), mandarin fish (*Siniperca chuatsi*), and stone moroko (*Pseudorasbora parva*) are commonly co-cultured with river crabs. However, research on the ecological impact of the stone moroko on river crab polyculture ponds remains limited. This study investigated the impacts of two polyculture systems involving river crabs on the composition of sediment microbial communities—specifically bacteria, fungi, and protists—in aquaculture pond environments. The results indicated that, compared to polyculture with mandarin fish and silver carp, the introduction of the stone moroko significantly altered the diversity, composition, co-occurrence networks, stability of these microbial communities, and environmental factors of the pond sediment. The polyculture of river crabs with mandarin fish, silver carp, and the stone moroko shows significant potential in enhancing the stability of sediment bacterial, fungal, and protistan communities and improving resource use efficiency in aquaculture, representing a viable aquaculture model with good ecological benefits.

## 1. Introduction

Globally, aquaculture is transitioning towards sustainable development practices to address critical challenges including climate change, population growth, and environmental degradation [1,2]. Integrated aquaculture, centered on multi-species polyculture, enhances resource utilization efficiency, environmental stability, and overall productivity, through establishing mutualistic interactions among species [3,4]. This production pattern not only increases economic returns, but also promotes balanced and resilient aquaculture ecosystems [3,4]. The Chinese mitten crab (*Eriocheir sinensis*) holds significant economic and cultural value in China, with its 2023 production reaching 888,629 tons and representing approximately 16.7% of the total freshwater crustacean production [5,6,7,8]. Pond aquaculture is the dominant production pattern for river crabs, primarily practiced in the middle and lower reaches of China’s Yangtze River Basin [8,9,10,11]. However, in traditional pond monoculture for river crabs, low utilization efficiencies lead to significant accumulation of unconsumed feed in sediments, resulting in deterioration of the aquaculture environment [12,13,14]. Consequently, current farming practices frequently co-culture river crabs with other species [9,10,11,15,16]. Evaluating the specific impacts of different co-cultured species on pond ecosystems is particularly important for optimizing and improving the pond production pattern for river crabs.

To enhance productivity and environmental stability, river crabs are typically co-cultured with silver carp (*Hypophthalmichthys molitrix*) and mandarin fish (*Siniperca chuatsi*) [15,16]. Silver carp improve water quality by filter-feeding on detritus and phytoplankton, while mandarin fish control disease vectors through predation on wild fish and optimize feed utilization efficiency [17,18,19,20]. With recent advances in aquaculture technology, the stone moroko (*Pseudorasbora parva*) has been experimentally introduced into river crab polyculture ponds. The stone moroko, a small omnivorous fish, predominantly consumes a diet comprising algae, plankton, and detrital organic matter [21]. The stone moroko is recognized as a high-quality aquatic product, due to its rapid growth rate and tender flesh, contributing to its strong consumer preference [21]. Preliminary studies indicate that *P. parva* significantly enhances microbial community diversity and stability in the water column [22]. However, its ecological impacts on pond sediment and benthic microbes remain uncharacterized. Sediments function not only as carriers for the migration and transformation of biogenic elements, but also as critical reservoirs and biogeochemical buffers, playing a role in regulating and mitigating sudden changes in water quality [23,24,25]. Concurrently, sediment microorganisms mediate biogeochemical processes at the sediment–water interface, profoundly influencing elemental cycling [26,27,28]. Therefore, elucidating the impact of the stone moroko on the benthic environment and microbial communities in river crab co-culture systems becomes particularly important.

In aquatic ecosystems, complex and multifaceted interactions occur among bacteria, fungi, and protists [29,30,31,32]. Fungi and protists have the capability to decompose complex organic substances that are difficult for bacteria to degrade, thereby modulating bacterial community dynamics [29,30,31,32]. Simultaneously, some heterotrophic protists can alter the composition of bacterial communities by preying on bacteria, while some autotrophic protists influence bacterial communities by providing shelter and nutrients [33,34]. Different microbial groups interact intensively through predation, mutual symbiosis, and competition [35,36]. Hence, in this study, we introduced the stone moroko into the pond polyculture of river crabs, silver carp, and mandarin fish, and conducted a comparative analysis of the impact of the stone moroko on benthic bacterial, fungal, and protistan communities, as well as on the physicochemical properties of sediments. The aim of this study is to establish a theoretical framework and provide empirical data for optimizing river crab polyculture models and achieving sustainable river crab production.

## 2. Materials and Methods

### 2.1. Experimental Location and Design

**Figure 1:**
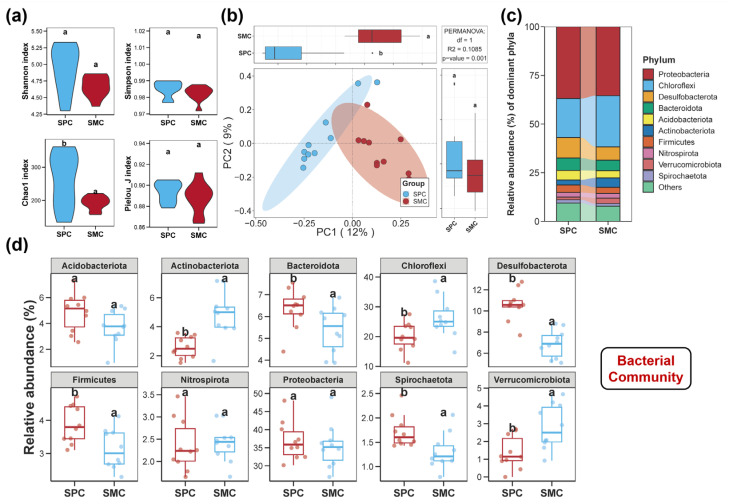
Diversity and composition of the bacterial community in pond sediment. (**a**) Differences in the alpha diversity indices of sediment bacterial community between the SPC and SMC groups. (**b**) Differences in the beta diversity of sediment bacterial community between the SPC and SMC groups assessed by principal coordinates analysis (PCoA). (**c**) The bacterial community composition in pond sediment at the phylum level. (**d**) The top ten dominant bacterial phyla in pond sediment and the differences in their relative abundances between the SPC and SMC groups. Different lowercase letters denote significant intergroup differences (SPC vs. SMC) for each indicator (*p* < 0.05).

This research was conducted at an aquaculture farm in Changzhou, Jiangsu Province, China (longitude 119.82° E, latitude 31.99° N). Two different polyculture ponds were established, each with an area of 0.87 hectares. One pond was a polyculture of the Chinese mitten crab with the mandarin fish (*Siniperca chuatsi*) and silver carp (*Hypophthalmichthys molitrix*), referred to as SMC (Figure 1). The other pond was stocked with a polyculture system consisting of the Chinese mitten crab, mandarin fish, silver carp, and stone moroko, hereafter abbreviated as SPC. During the farming period, both ponds employed the same cultivation methods and management practices. The feeding frequency for the river crabs was once daily. The experimental diet comprised a formulated mixture of commercial pellet feed (supplied by Changzhou Xinbei Menghe Hongzhi Fish Aquaculture Farm, Changzhou, China), fresh frozen trash fish, and fermented corn, at a fixed ratio of 1:7:2 (by weight). The farming period lasted for 215 days, from March 15th to October 15th.

All experimental animals (including the Chinese mitten crab, silver carp, mandarin fish, and stone moroko) were identified by experienced taxonomists using traditional morphological methods. The identification was based on, but not limited to, external morphological characteristics. The taxonomic criteria primarily referred to authoritative references [37,38,39,40]. Only after all individuals were confirmed to be the target species, were they utilized in this study.

### 2.2. Experimental Sample Collection and Methods

Starting from August, crab farming enters a critical period, especially in September. This period represents a critical window for the fattening and growth of river crabs. Therefore, the specific date for sampling was set for 3 September 2023. Ten sampling points were systematically established in each pond to collect ten replicate sediment samples. Sediment samples were collected from each sampling point, using a sediment sampler. Each replicate was subdivided for concurrent DNA extraction and physicochemical analysis. After sampling, the grouped sediment samples were immediately stored at −80 °C.

### 2.3. Characterization of Sediment Physicochemical Properties

Prior to analysis, sediment samples were lyophilized (Freeze Dryer Model: DG-65Z04-10AR; Haier Biomedical, Qingdao, China), and subsequently ground into powder form. The total sulfur (TS), total nitrogen (TN), and total carbon (TC) concentrations were determined using a Vario EL Cube Elemental Analyzer (Elementar Analysensysteme GmbH, Frankfurt, Germany). The ammonia, nitrate, and nitrite contents in the samples were determined using the spectrophotometry methods [41,42]. The phosphate and total phosphorus (TP) levels were determined by SMT protocol, according to the modified Williams protocol [43]. Prior to measurement, ammonia, nitrate, nitrite, and phosphate in the samples were extracted using a 2 mol/L potassium chloride (KCl) solution [41,42].

### 2.4. Extraction and Sequencing of Microbial DNA from Pond Sediments

Microbial DNA was extracted from the pond sediment samples through the E.Z.N.A.^®^ Soil DNA Kit (Omega Bio-tek, Norcross, GA, USA). Subsequent amplification targeted the bacterial 16S rRNA V3-V4 region (primers 338F/806R [44]), the fungal ITS1-ITS2 region (primers ITS1F/ITS2R [45]), and the protistan 18S rRNA V4 region (primers TAReuk454FWD1/TAReukREV3 [46]). The amplification products were separated by 2% agarose gel electrophoresis and purified using the AxyPrep DNA Gel Extraction Kit (Axygen Biosciences, Union City, CA, USA). Additionally, the NEXTFLEX Rapid DNA-Seq Kit (Bioo Scientific, Austin, TX, USA) was used to construct the NovaSeq library.

Raw sequencing reads were processed using FASTP for quality control and FLASH for assembly, with a minimum overlap of 10 bp and a maximum mismatch error rate of 2% [47,48]. After removing duplicates, the DADA2 algorithm in QIIME 2 was applied to detect insertion–deletion and substitution mutations, and to define Amplicon Sequence Variants (ASVs) [49]. Paired-end reads were trimmed and filtered under a threshold of maxEE ≤ 2. Taxonomic classification of bacterial, fungal, and protistan ASVs was performed using the Silva SSU132, UNITE, and PR2 databases, respectively [50,51,52].

### 2.5. Bioinformatics Analysis and Statistics

The Shannon, Simpson, Chao1, and Pielou_J indices were used to assess the diversity and evenness of the bacterial, fungal, and protistan communities in pond sediments. Principal Coordinates Analysis (PCoA) based on Bray–Curtis distance was employed to visualize differences in bacterial, fungal, and protistan communities between the SPC and SMC groups, with the significance of these differences evaluated using Permutational Multivariate Analysis of Variance (PERMANOVA). Differences in the physicochemical properties of pond sediment between the SPC and SMC groups were analyzed using an independent samples *t*-test. Wilcoxon rank-sum tests were used to compare differences in the relative abundances of the top ten most abundant bacterial, fungal, and protistan phyla and genera between the SPC and SMC groups. The environmental adaptability of microbial communities between groups was assessed based on habitat niche breadth and dispersal capability [53,54]. Co-occurrence networks were constructed based on the Spearman correlation matrix (Spearman’s r > 0.6 and *p*-value < 0.05) to evaluate the intrinsic interactions within the microbial communities. Distance-based redundancy analysis (db-RDA) was performed to assess the relationships between sediment microbial communities and environmental factors across the experimental groups. The aggregated boosted tree (ABT) model was utilized to assess the contribution of environmental factors to community diversity and to identify the primary drivers of community variation [55].

## 3. Results

### 3.1. Diversities of the Sediment Bacterial, Fungal, and Protistan Communities

**Figure 2:**
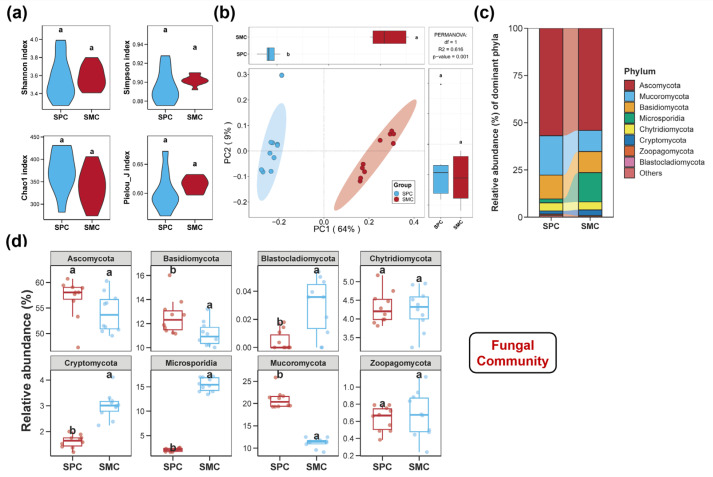
Diversity and composition of the fungal community in pond sediment. (**a**) Differences in the alpha diversity indices of sediment fungal community between the SPC and SMC groups. (**b**) Differences in the beta diversity of sediment fungal community between the SPC and SMC groups assessed by principal coordinates analysis (PCoA). (**c**) The fungal community composition in pond sediment at the phylum level. (**d**) The top ten dominant bacterial phyla in pond sediment and the differences in their relative abundances between the SPC and SMC groups. Different lowercase letters denote significant intergroup differences (SPC vs. SMC) for each indicator (*p* < 0.05).

**Figure 3:**
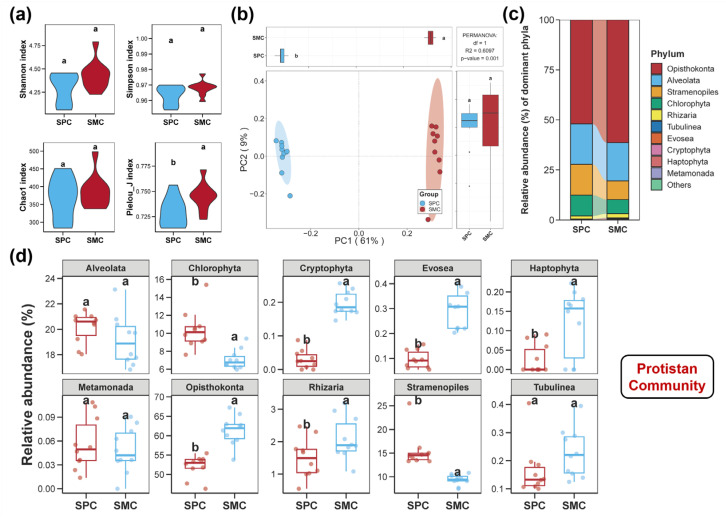
Diversity and composition of the protistan community in pond sediment. (**a**) Differences in the alpha diversity indices of sediment protistan community between the SPC and SMC groups. (**b**) Differences in the beta diversity of sediment protistan community between the SPC and SMC groups assessed by principal coordinates analysis (PCoA). (**c**) The protistan community composition in pond sediment at the phylum level. (**d**) Differences in the relative abundances of the top ten most abundant protistan phyla within pond sediment between the SPC and SMC groups. Different lowercase letters denote significant intergroup differences (SPC vs. SMC) for each indicator (*p* < 0.05).

The diversities of bacterial, fungal, and protistan communities in pond sediments are illustrated in Figure 1, Figure 2 and Figure 3. There were no notable differences in the Shannon, Simpson, and Pielou_J indices of the sediment bacterial community between the SPC and SMC groups (Figure 1a, *p* > 0.05). However, the SPC group obviously increased the Chao1 index of the bacterial community in pond sediment (Figure 1a, *p* < 0.05). Regarding the fungal community diversity in pond sediment, neither Shannon, Simpson, Pielou_J, nor Chao1 indices differed significantly between the two groups (Figure 2a, *p* > 0.05). For the protistan community in pond sediment, no remarkable differences in the Shannon, Simpson, and Chao1 indices between the SPC and SMC groups were observed (Figure 3a, *p* > 0.05), but the SPC group remarkably reduced the Pielou_J index of the protistan community (Figure 3a, *p* < 0.05). Additionally, PCoA analysis further revealed significant compositional differences in sediment bacterial, fungal, and protistan communities between the SPC and SMC groups (Figure 1b, Figure 2b and Figure 3b, *p* < 0.05).

### 3.2. Compositions of the Sediment Bacterial, Fungal, and Protistan Communities

In the pond sediments, a total of 50 bacterial phyla were identified. The top ten most abundant phyla were Proteobacteria, Chloroflexi, Desulfobacterota, Bacteroidota, Acidobacteriota, Actinobacteriota, Firmicutes, Nitrospirota, Verrucomicrobiota, and Spirochaetota (Figure 1c). Compared to the SMC group, the SPC group substantially increased the relative abundance of Desulfobacterota, Bacteroidota, Firmicutes, and Spirochaetota, while decreasing the relative abundance of Chloroflexi, Actinobacteriota, and Verrucomicrobiota (Figure 1d, *p* < 0.05).

For the fungal community, eight phyla were detected in pond sediment, ranked by relative abundance, as follows: Ascomycota, Mucoromycota, Basidiomycota, Microsporidia, Chytridiomycota, Cryptomycota, Zoopagomycota, and Blastocladiomycota (Figure 2c). The SPC group exhibited dramatically higher relative abundances of Mucoromycota and Basidiomycota, but lower relative abundances of Cryptomycota and Blastocladiomycota, compared to the SMC group (Figure 2d, *p* < 0.05).

Among protists, 22 phyla were identified in pond sediment, with the top 10 most abundant being Opisthokonta, Alveolata, Stramenopiles, Chlorophyta, Rhizaria, Tubulinea, Evosea, Cryptophyta, Haptophyta, and Metamonada (Figure 3c). The SPC group showed considerably higher abundances of Stramenopiles and Chlorophyta, but lower relative abundances of Opisthokonta, Rhizaria, Evosea, Cryptophyta, and Haptophyta, relative to the SMC group (Figure 3d, *p* < 0.05).

### 3.3. Co-Occurrence Networks and Stabilities of the Sediment Bacterial, Fungal, and Protistan Communities

**Figure 4:**
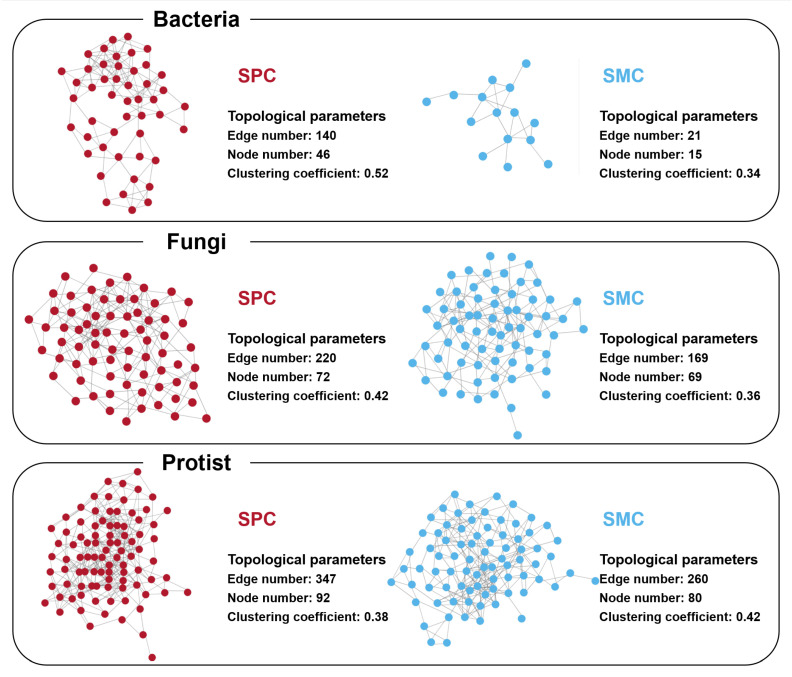
Co-occurrence networks of the bacterial, fungal, and protistan communities in pond sediment.

Co-occurrence networks of bacterial, fungal, and protistan communities in pond sediment were established to reflect the microbial interactions (Figure 4). The co-occurrence network of the bacterial community in the SPC group displayed greater complexity (46 nodes, 140 edges; clustering coefficient = 0.52) compared to the SMC group (5 nodes, 21 edges; clustering coefficient = 0.34) (Figure 4). Similarly, the fungal co-occurrence network showed higher connectivity in the pond sediment of SPC group (72 nodes, 220 edges; clustering coefficient = 0.42) than in that of SMC group (69 nodes, 169 edges; clustering coefficient = 0.36) (Figure 4). The most extensive and complex co-occurrence networks were observed for protists, with sediment protistan co-occurrence network in SPC group exhibiting 92 nodes and 347 edges, while that in SMC group contained 80 nodes and 260 edges (Figure 4).

**Figure 5:**
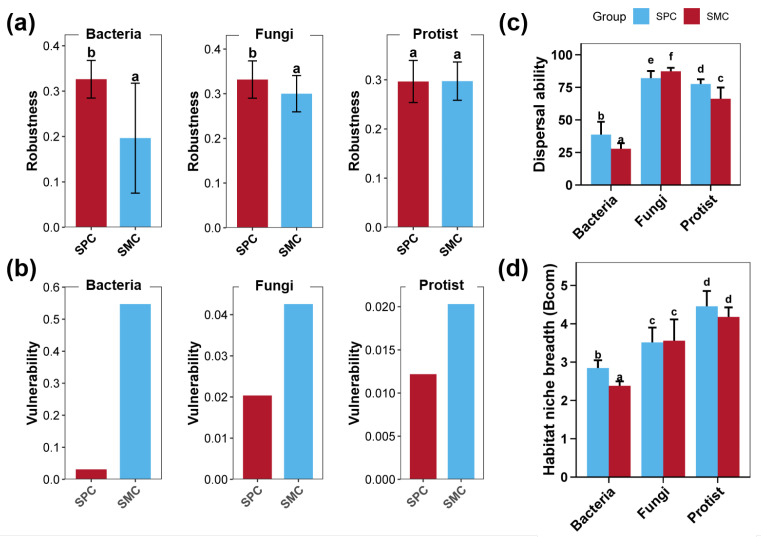
The stability of bacterial, fungal, and protistan communities in pond sediment. (**a**) Differences in the robustness index for the bacterial, fungal, and protistan co-occurrence networks in pond sediment between the SPC and SMC groups. (**b**) Differences in the vulnerability index for the bacterial, fungal, and protistan co-occurrence networks in pond sediment between the SPC and SMC groups. (**c**) Differences in the dispersal abilities for the bacterial, fungal, and protistan communities in pond sediment between the SPC and SMC groups. (**d**) Differences in the habitat niche breadths for the bacterial, fungal, and protistan communities in pond sediment between the SPC and SMC groups. Different lowercase letters denote significant intergroup differences (SPC vs. SMC) for each indicator (*p* < 0.05).

The stability of bacterial, fungal, and protistan communities in pond sediment was assessed through robustness, vulnerability, and habitat niche breadth indices (Figure 5). The bacterial and fungal co-occurrence networks within pond sediment in the SPC group exhibited dramatically higher robustness than those in the SMC group (Figure 5a, *p* < 0.05), while the robustness of the protistan co-occurrence network showed no marked difference between the two groups (Figure 5a, *p* > 0.05). The co-occurrence networks of sediment bacterial, fungal, and protistan communities exhibited significantly lower vulnerability in the SPC group compared to the SMC group (Figure 5b). Dispersal ability displayed group-specific patterns, with bacterial and protistan communities showing substantially higher values in the pond sediment of the SPC group, whereas fungal communities exhibited the opposite trend, with significantly reduced dispersal ability in the SPC group (Figure 5c, *p* < 0.05). Regarding habitat niche breadth, only sediment bacterial communities in the SPC group exhibited broader niches than those in SMC group (Figure 5d, *p* < 0.05), while fungal and protistan communities displayed similar niche breadths between the SPC and SMC groups (Figure 5d, *p* > 0.05).

### 3.4. Environmental Factors in Pond Sediment and Their Correlations with Microbial Communities

**Figure 6:**
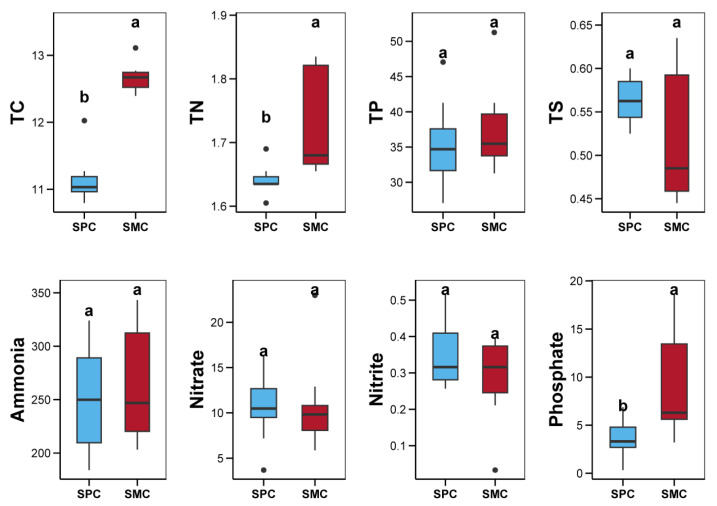
Differences in the total carbon (TC, μg/mg), total nitrogen (TN, μg/mg), total phosphorus (TP, μg/g), total sulfur (TS, μg/mg), ammonia (μg/g), nitrate (μg/g), nitrite (μg/g), and phosphate (μg/g) within pond sediment, between the SPC and SMC groups. Different lowercase letters denote significant intergroup differences (SPC vs. SMC) for each indicator (*p* < 0.05).

**Figure 7:**
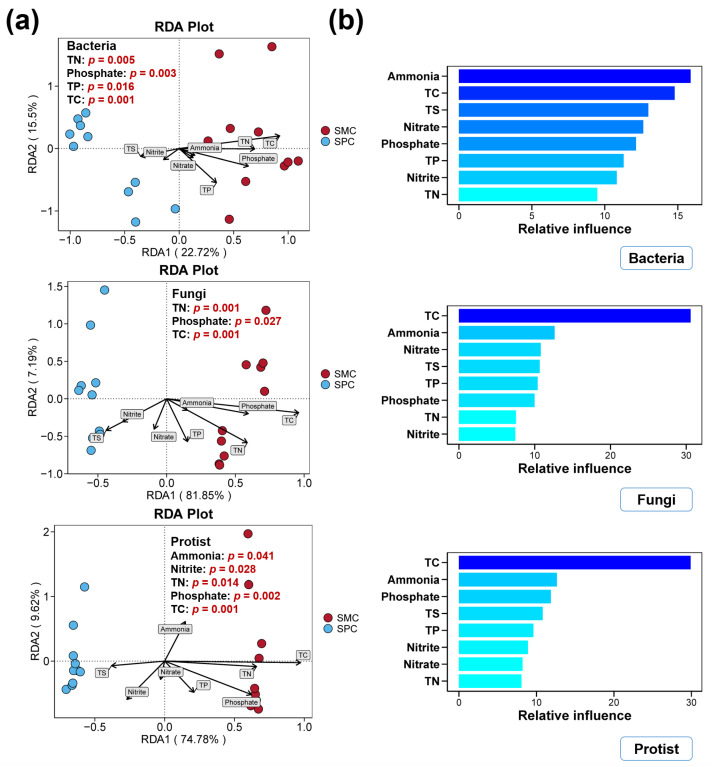
Associations between environmental factors and sediment microbial communities. (**a**) Distance-based redundancy analysis (db-RDA) exhibiting associations between sediment parameters and microbial communities in SPC and SMC groups. (**b**) Aggregated boosted tree (ABT) analysis quantifying the relative influence of environmental variables on the microbial communities in pond sediment.

The concentrations of TC, TN, TP, TS, ammonium, nitrate, nitrite, and phosphate in pond sediments between the SPC and SMC groups are shown in Figure 6. The TC, TN, and phosphate levels in pond sediment of the SPC group were obviously lower than those in the SMC group (*p* < 0.05, Figure 6). However, there were no remarkable differences between the SPC and SMC groups in terms of TP, TS, ammonium, nitrate, and nitrite contents in the sediment (*p* > 0.05, Figure 6). As shown in Figure 7, considerable correlations between the environmental factors and the microbial communities were identified (*p* < 0.05). The concentrations of TC, TN, TP, and phosphate in pond sediment showed remarkable correlations with bacterial communities (*p* < 0.05, Figure 7a). Similarly, TC, TN, and phosphate levels were considerably associated with sediment fungal communities, while TC, TN, ammonium, nitrite, and phosphate contents exhibited notable correlations with protistan communities (*p* < 0.05, Figure 7a). ABT analysis revealed that the TC and ammonium levels in pond sediment were key environmental factors influencing sediment bacterial, fungal, and protistan communities (Figure 7b). Notably, for the sediment fungal and protistan communities, TC made the largest relative contribution, substantially exceeding that of ammonium and other environmental factors (Figure 7b).

## 4. Discussion

### 4.1. Alterations in the Diversities and Compositions of the Sediment Bacterial, Fungal, and Protistan Communities

Previous studies have demonstrated that, compared to the polyculture of river crabs with mandarin fish and silver carp, stone moroko addition substantially altered microbial composition but had limited effects on overall diversity metrics [22]. In this study, the addition of the stone moroko had a substantial impact on the composition of the microbial communities in pond sediment, but its effect overall on microbial diversity was comparatively limited. Specifically, it considerably increased only bacterial community richness, while reducing protistan community evenness. Regarding community composition, the SPC group significantly affected all fungal phyla and most of the dominant (top 10 in relative abundance) bacterial and protistan phyla. Bacterial phyla including Firmicutes, Bacteroidota, Acidobacteriota, Chloroflexi, Verrucomicrobiota, and Actinobacteriota play critical roles in biogeochemical cycling by driving the transformation of carbon, nitrogen, phosphorus, and sulfur [56,57,58,59,60,61,62]. Fungi possess immense metabolic diversity, and actively contribute to the mineralization of humic compounds, making them an important component in the transformation of organic matter and energy cycling in aquatic ecosystems [63,64,65]. Protists such as Stramenopiles can function both as consumers and producers, playing a crucial role in the carbon and mineral cycles [66,67,68,69,70]. Therefore, the pronounced differences in microbial community compositions under different polyculture methods of river crabs might suggest a profound impact on the matter cycling mediated by bacteria, fungi, and protists in pond sediment. Previous studies have established that polyculture operations can induce changes in microbial communities via interspecific interactions and bioturbation [71,72]. Accordingly, the pronounced shifts in microbial composition observed in this study might reflect ecological changes resulting from the introduction of the stone moroko.

### 4.2. Changes in the Microbial Co-Occurrence Network and Stability

Microbial co-occurrence networks reveal complex interactions among microorganisms [73]. The number of nodes and edges in these networks reflects the connectivity and complexity of microbial communities [74]. Microbial communities with complex structure and higher connectivity often indicate greater ecological niche overlap and functional redundancy among bacteria, traits that contribute to microbial community stability and resilience under environmental changes [19,75]. The co-occurrence networks of bacterial, fungal, and protistan communities in the SPC group demonstrated a greater number of nodes and edges compared to those in the SMC group, indicating more complex and stable communities. Additionally, the robustness indices of the sediment bacterial and fungal communities in the SPC group increased markedly compared to the SMC group, while their vulnerability decreased. These results collectively suggested that the addition of the stone moroko in the polyculture of river crabs with mandarin fish and silver carp led to more complex and stable bacterial, fungal, and protistan communities in pond sediment [76,77]. Further analysis was conducted on the habitat niche breadths of bacterial, fungal, and protistan communities in pond sediment. The breadth of a microbial habitat niche serves as an indicator of community stability; a narrower niche breadth implies constrained metabolic versatility and lower environmental adaptability [78]. Compared to the SMC group, the habitat niche breadth of the bacterial community in the SPC group increased significantly, further indicating the beneficial effect of introducing the stone moroko on the stability of microbial communities in pond sediment.

### 4.3. Alterations in Environmental Factors and Their Correlations with Bacterial, Fungal, and Protistan Communities

Previous studies have found that different aquaculture production patterns can lead to changes in environmental microbial communities, and these changes are almost always driven by variations in environmental factors [20,79,80,81,82]. Similarly, our research found that the incorporation of the stone moroko, compared to the polyculture of river crabs with mandarin fish and silver carp, considerably reduced the TC, TN, and phosphate contents in pond sediment, and altered the composition, diversity, and co-occurrence network of bacterial, fungal, and protistan communities. Meanwhile, the bacterial, fungal, and protistan communities were substantially correlated with the levels of TC, TN, and phosphates in pond sediment, with TC particularly identified as a key environmental factor influencing these communities. The TC plays a crucial role in determining microbial diversity and biomass by providing the necessary energy sources and substrates for microbial metabolism [83]. Nitrogen and phosphorus, as essential nutrients for microbial reproduction, can regulate microbial growth [84,85]. Alterations in phosphorus concentration can further drive shifts in the composition of fungal and protistan communities [86,87,88,89,90]. Previous research has similarly demonstrated the significant correlations between the concentrations of TC, TN, and phosphates and the composition of microbial communities in aquatic environments [79,80,91]. Therefore, in this study, variations in microbial community composition, diversity, co-occurrence networks, and stability are likely driven by polyculture practices-mediated changes in environmental conditions, especially TC level.

Furthermore, it is noteworthy that the substantial feed input in pond aquaculture leads to the accumulation of considerable aquaculture waste in the sediment, including uneaten feed, feces, and other organic debris [92,93,94,95,96,97]. The accumulation of aquaculture waste in the sediments poses a risk of endogenous water pollution, which presents a potential threat to the pond ecosystem and the surrounding aquatic environment [98,99]. The co-cultivation of river crabs with mandarin fish, silver carp, and the stone moroko, resulting in the reductions in TC, TN, and phosphate contents, might indicate an improvement in the utilization efficiency of aquaculture resources and a decrease in aquaculture waste. This is crucial for maintaining a good farming environment and developing sustainable aquaculture.

## 5. Conclusions

Overall, compared to the polyculture system containing only river crab, mandarin fish, and silver carp, the addition of the stone moroko remarkedly impacted the diversity, composition, and co-occurrence network structure of bacterial, fungal, and protistan communities in pond sediment. The polyculture system containing river crab, mandarin fish, silver carp, and the stone moroko increased bacterial community richness while reducing protistan community evenness, and altered the relative abundances of all fungal phyla and most of the dominant (top 10 in relative abundance) bacterial and protistan phyla. The stone moroko also enhanced the complexity and stability of bacterial, fungal, and protistan communities, as revealed by the co-occurrence networks and habitat niche breadth. The addition of the stone moroko led to notably decreased TC, TN, and phosphates levels in pond sediment, which might indicate an improvement in resource utilization efficiency in pond aquaculture, thereby preventing the environmental risks associated with excessive nutrient accumulation in sediment during cultivation. Additionally, the sediment TC, TN, and phosphates concentrations were pronouncedly correlated with the microbial communities, with TC being the main contributor driving changes in microbial communities. The polyculture of river crabs with mandarin fish, silver carp, and the stone moroko is a viable aquaculture model with favorable ecological benefits. Our research established a theoretical framework and provided empirical data for optimizing river crab polyculture models and achieving sustainable river crab production.

However, this study still has certain limitations. On one hand, it did not evaluate production indicators such as the yield of aquatic animals. On the other hand, samples were collected and analyzed at only one representative time point during the cultivation process, which means the research results cannot reflect the dynamic changes throughout the entire cultivation cycle. Therefore, it is essential to conduct continuous monitoring over multiple years to understand the dynamic changes in pond environmental microorganisms over a longer, complete production cycle. Conducting long-term and multi-season studies is indispensable for extending these microbiological research findings to broader aquacultural production.

## Data Availability

The raw data supporting the conclusions of this study are available from the corresponding author upon reasonable request.
